# Fuzzy evaluation on the integrated benefit of regulated deficit irrigation for pear‐jujube tree based on information entropy

**DOI:** 10.1002/fsn3.3313

**Published:** 2023-03-13

**Authors:** Weihua Guo, Yun Gao

**Affiliations:** ^1^ College of Agricultural Engineering Hohai University Nanjing China; ^2^ State Key Laboratory of Hydrology‐Water Resources and Hydraulic Engineering Hohai University Nanjing China

**Keywords:** analytic hierarchy process, fuzzy evaluation, information entropy, integrated benefit, pear‐jujube tree (*Zizyphus jujube Mill.*), regulated deficit irrigation

## Abstract

Based on the experimental data of regulated deficit irrigation at different growth stages on field pear‐jujube tree (*Zizyphus jujube Mill.*) during 2005–2007 in Northwest China, the regulated deficit irrigation integrated benefits (RDIIB) with different water deficit treatments at different growth stages were evaluated and classified using a model. The results in 2005–2006 showed that the RDIIB under single‐stage water deficit at fruit maturity stage were better than the other treatments, and the best RDIIB were gained under moderate (IVSD) or severe deficit (IVMD) at fruit maturity stage. The results in 2006–2007 also indicated that the four double‐stage water deficit schemes have the better RDIIB, and the best scheme was severe water deficit at bud burst to leafing stage plus moderate water deficit at fruit maturity stage. The RDIIB evaluation model based on information entropy method provided the reliable technical guidance for the optimal RDI scheme of pear‐jujube tree.

## Graphical abstract

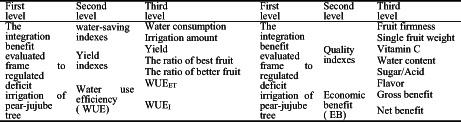

## 1 INTRODUCTION

Regulate deficit irrigation (RDI), as an important physiological water‐saving technique, has a great deal of researches been applied on fruit trees since it was put forward (Mitchell et al., 1984). It was tested to save irrigation water and improve fruit quality significantly without reducing or slightly increasing the fruit yield, therefore, it was obviously increasing water use efficiency (WUE) and economic benefit (EB), However, the RDI technique should be implemented in the suitable water deficit stage, degree and duration, and different water deficit had different effect on the fruit yield, quality, WUE and EB (Ahmed et al., 2007; Costa et al., 2007; Cui et al., 2008; Iniesta et al., 2008). Even under the same water deficit pattern, the effect of RDI on above‐mentioned benefit parameters were also different, which has a great impact on the optimization of RDI technology. Therefore, it may be a feasible way to take a comprehensive consideration of most information about RDI on fruit yield, quality, WUE and EB using the advanced mathematical principles to evaluate regulate deficit irrigation integration benefits (RDIIB) scientifically, and optimize the RDI scheme for the local orchards.

Historically, information theory was developed to find fundamental limits on compressing and reliably storing and communicating data. Since its inception, it has broadened to find applications in many other areas to describe the uncertainty, a key measure of information in the theory is known as entropy, which is usually expressed by the average number of bits needed for storage or communication. Intuitively, entropy quantifies the uncertainty involved when encountering a random variable (Shannon, 1948). The information entropy weight is calculated through the assessment of matrix in condition that all the estimating parameters were given. Each parameter has a figure in the sense of the relative intensity, which indicates the amount of the useful information supplied by the given parameter, in ways of information theory; it also represents how much useful information provided in the evaluation with such a parameter. Therefore, it may provide more important objective basis to calculate the parameter's weight using information entropy method (Jin, 2007; Shi et al., 2008).

Tang et al. (2000) put forward an optimization model with entropic coefficients for management in irrigation water resources, which take a comprehensive consideration of the economic benefit, social demand and ecological benefit of sustainable agricultural development. Entropic coefficients method for comprehensive evaluation of crop varieties was also discussed with considering the crop traits to avoid experimental deviation induced by emphasizing some crop traits but neglecting other characteristics (Tang & Wang, 2002). Li et al. (2006) evaluated the operation status of irrigation district using information entropy method, and compared with the results using gray system evaluation methods, the results showed that the array orders by both methods were similar. Reservoir flood control operation was also investigated using multi‐objective schemes optimum with the combination of the information entropy theory and fuzzy optimization model, the subjective weight were taken into account by the linear combination of the multi‐objective weights, and received the optimal membership degree of optimal scheme, as a result, the problem of multi‐objective weight allocation was solved reasonably (Zhou et al., 2007). Based on the data of irrigation district reconstruction and water‐saving reform evaluation, and combined with information entropy weight method and the experts method, multi‐objective fuzzy theory and methods were also used to evaluate and rank the effect of water‐saving reform in some irrigation districts of the Yellow River Basin by Zhang et al. (2008). This method was also widely used in the evaluation of labor market interventions (Walker, 2007), evaluation on economic efficiency of agricultural land (Li & Diao, 2008) and network traffic Performance (Lall et al., 2006). The fuzzy‐optimized, multi‐objective, and multilevel comprehensive evaluation model for the comprehensive benefits of water resources in irrigation area was established on the premise of improving the weight vector decision method of different layers (Feng & Xu, 2009).

Although many above‐mentioned literatures are related to information entropy theory to agricultural water management, there is neither reports on comprehensive consideration of different water deficit treatment effects on fruit yield, quality, WUE, and EB under conditions of RDI, nor application of information entropy in fruit tree RDIIB evaluation.

Jujube (*Zizyphus jujube Mill*.) originated from China and has been planted for more than 5000 years. Now the planting area of jujube in China is about 30,000 ha, with total yield of 600 million kg. It is mainly cultivated in northern China, however, inappropriate irrigation may result in waste of water resources and poor fruit quality (Cui et al., 2009). Therefore, the implement of suitable RDI schemes can save much irrigation water and improve the economic efficiency of the orchard production. In this paper, the aim was to establish the RDIIB evaluation model of fruit tree based on information entropy, select optimal RDI scheme and provide guidance for developing high‐efficient, water‐saving, and sustainable fruit production in arid and semiarid regions.

## 2 MATERIALS AND METHODS

### 2.1 Experiment and measurements

The field experiments were continuously conducted during 2005–2007; the fruit tree characteristics, experimental site, and design were described in our published references (Cui et al., 2008, 2009). As supplementary of the 2 year's field experiment of single‐stage regulated deficit irrigation as described by Cui et al. (2008), two additional treatments with double‐stage water deficit were carried out in 2006 and 2007 (Table 1). Soil water content, fruit yield, fruit quality parameters, such as mean single fruit weight, mean single fruit volume, fruit water content, fruit figure index, fruit flavor, organic acid, vitamin C (V_C_), soluble protein, soluble solid content, and fruit firmness were measured, sugar/acid and rotten fruit percentage were calculated simultaneously. The measurement methods were the same as that followed by Cui et al. (2008). The gross economic benefit was calculated according to the actual market price and yield with different rank at harvesting stages; and the net economic benefit was gained by deducting irrigation water and labor inputs (pruning, flowers and fruit, irrigation manpower charge, etc.) and other inputs (fertilizers, pesticides etc.) from the gross economic benefits.

**TABLE 1:** Integration benefit evaluation frame of regulated deficit irrigation in pear‐jujube tree.

First level	Second level	Third level	First level	Second level	Third level
The integration benefit evaluated frame of regulated deficit irrigation in pear‐jujube tree	Water‐saving indexes	Water consumption	The integration benefit evaluated frame of regulated deficit irrigation in pear‐jujube tree	Quality indexes	Fruit firmness
		Irrigation amount			Single fruit weight
	Yield indexes	Yield			Vitamin C
		The ratio of best fruit			Water content
		The ratio of better fruit			Sugar/Acid
	Water use efficiency (WUE)	WUE_ET_			Flavor
		WUE_I_		Economic benefit (EB)	Gross benefit
					Net benefit

### 2.2 RDI comprehensive benefits evaluation hierarchy model and parameters system

In general, integration benefit evaluation frame of RDI for pear‐jujube tree consists of the effect of different water deficit at different growing stages on water‐saving, fruit yield, fruit quality, WUE, and EB, especially as for the fruit quality parameters, six representative fruit quality parameters were selected through investigation, principal component analysis, and clustering analysis. Considering the connotation and representative of all the parameters systematically, integration benefit evaluation hierarchy frame of RDI (RDIIB) was established in Table 1.

### 2.3 The establishment of RDIIB model

#### 2.3.1 Decision‐making eigenvalue matrix

Suppose that multi‐objective decision‐making scheme set is *P* = (*P*_1_, *P*_2_, …, *P*_*N*_), object set is *A* = (*A*_1_, *A*_2_, …, *A*_*M*_), then decision‐making eigenvalue matrix with *n* as scheme and *m* as evaluation parameter is:

(1) $$X=\left[\begin{array}{cccc} x_{11}, & x_{12}, & \ldots & x_{1n}\\ x_{21}, & x_{22}, & \ldots & x_{2n}\\ \ldots & \ldots & \ldots & \ldots \\ x_{m1}, & x_{m2}, & \ldots & x_{\mathrm{mn}}, \end{array}\right]=\left(x_{\mathrm{ij}}\right)\;\left(i=1,2,\ldots ,m;\;j=1,2,\ldots ,n\right)$$

#### 2.3.2 Relative membership degree matrix

In the evaluation frame as shown in Table 2, there are efficiency and cost parameters, in which the parameter will reflect the relative importance for different treatments after standardization. To avoid the minimum efficiency parameter and the maximum cost‐based parameter change to zero after range transformation, an improved range transformation formula is used as follows:

**TABLE 2:** Basic indices data of regulated deficit irrigation in different growth stages of pear‐jujube tree.

Year	Growth stages	Treatment	Water consumption (mm)	Irrigation amount (m^3^·hm^−2^)	Yield (kg F·hm^−2^)	The ratio of best fruit (%)	The ratio of better fruit (%)	WUE_ET_ (kg m^−3^)	WUE_I_ (kg m^−3^)
2005		T_1_ (CK)	605.20	3600	34336.50	65	35	5.67	9.53
	Bud burst to leafing stage (I)	T_2_ (SD)	516.37	2700	39352.50	70	30	7.62	14.57
		T_3_ (MD)	560.07	3150	39187.50	68	32	7.00	12.43
		T_4_ (LD)	575.8	3300	30112.5	72	28	5.23	9.12
	Flowering to fruit set stage (II)	T_5_ (SD)	522.08	2700	23,760	62	38	4.55	8.80
		T_6_ (MD)	561.35	3150	31,350	67	33	5.58	9.95
		T_7_ (LD)	581.04	3300	27,720	70	30	4.77	8.39
	Fruit growth stage (III)	T_8_ (SD)	519.96	2700	27307.5	73	27	5.25	10.11
		T_9_ (MD)	560.96	3150	28132.5	75	25	5.02	8.93
		T_10_ (LD)	580.97	3300	36,300	77	23	6.25	10.99
	Fruit maturity stage (IV)	T_11_ (SD)	521.5	2700	40342.5	76	24	7.74	14.93
		T_12_ (MD)	562.25	3150	38659.5	80	20	6.88	12.27
		T_13_ (LD)	576.87	3300	37207.5	77	23	6.45	11.26
2006		T_1_ (CK)	537.88	3600	36,465	62	38	6.78	10.12
	Bud burst to leafing stage (I)	T_2_ (SD)	442.25	2700	48097.5	60	40	10.88	17.8
		T_3_ (MD)	489.46	3150	41332.5	65	32	7.10	13.11
		T_4_ (LD)	506.04	3300	30227.5	68	22	5.98	9.17
	Flowering to fruit set stage (II)	T_5_ (SD)	446.6	2700	35103.75	63	37	7.86	12.99
		T_6_ (MD)	489.88	3150	29,535	65	35	6.03	9.37
		T_7_ (LD)	505.23	3300	31886.25	66	34	6.31	9.65
	Fruit growth stage (III)	T_8_ (SD)	441.37	2700	32051.25	70	30	7.26	11.86
		T_9_ (MD)	491.3	3150	29,040	72	28	5.91	9.21
		T_10_ (LD)	504.32	3300	36712.5	75	25	7.28	11.11
	Fruit maturity stage (IV)	T_11_ (SD)	449.89	2700	40342.5	76	24	8.97	14.93
		T_12_ (MD)	490.7	3150	40012.5	73	27	7.88	12.7
		T_13_ (LD)	505.7	3300	37207.5	80	20	7.36	11.26
2007		T_1_ (CK)	620.56	2700	37009.50	57	43	5.96	13.71
	Bud burst to leafing stage (I)	T_2_ (SD)	562.65	1800	42108.00	60	40	7.48	23.39
		T_3_ (MD)	608.74	2250	40260.00	62	38	6.61	17.89
		T_4_ (LD)	606.74	2400	31911.00	65	35	5.26	13.30
	Flowering to fruit set stage (II)	T_5_ (SD)	546.06	1800	28545.00	72	28	5.23	15.86
		T_6_ (MD)	598.95	2250	30277.50	70	30	5.06	13.46
		T_7_ (LD)	597.76	2400	33247.50	74	26	5.56	13.85
	Fruit maturity stage (IV)	T_11_ (SD)	575.16	1800	37801.50	76	24	6.57	21.00
		T_12_ (MD)	591.33	2250	39022.50	80	20	6.60	17.34
		T_13_ (LD)	571.92	2400	35062.50	82	18	6.13	14.61

Year	Growth stages	Treat ‐ment	Fruit firmness (N·cm^−2^)	Vitamin C (Vc) (mg·100 g^−1^FW)	Single fruit weight(g)	Water content (%)	Sugar/acid	Flavor	The gross benefit (万￥·hm^−2^)	The net benefit (万￥·hm^−2^)
2005		T_1_ (CK)	5.85	43.59	32.28	64.35	32.42	7.40	8.86	8.11

Bud burst to leafing stage (I)	T_2_ (SD)	6.53	45.45	37.19	63.02	32.71	8.00	10.39	9.82

	T_3_ (MD)	7.64	45.6	34.59	65.13	60.83	7.80	10.25	9.63

T_4_ (LD)	7.93	43.27	32	61.79	87.23	7.50	8.02	7.35

Flowering to fruit set stage (II)	T_5_ (SD)	7.1	45.35	30.6	58.41	107.35	8.50	6.04	5.43

	T_6_ (MD)	7.21	42.93	27.65	61.03	75.46	8.20	8.16	7.47

T_7_ (LD)	7.16	43.51	31.57	66.07	60.82	7.70	7.32	6.62

Fruit growth stage (III)	T_8_ (SD)	7.42	41.95	30.52	57.26	106.48	9.10	9.74	9.08

	T_9_ (MD)	7.35	49.45	32.04	57.43	37.76	8.80	10.13	9.43

T_10_ (LD)	7.32	56.59	31.12	59.35	93.39	9.00	13.18	12.44

Fruit maturity stage (IV)	T_11_ (SD)	7.25	49.72	33.2	55.77	68.78	9.20	14.59	13.86

	T_12_ (MD)	7.65	48.92	34.07	58.01	79.82	9.40	14.23	13.49

T_13_ (LD)	7.74	49.44	34.07	60.52	57.33	9.50	13.51	12.77
2006		T_1_ (CK)	5.50	31.85	29.21	59.62	49.68	7.20	9.28	8.53
	Bud burst to leafing stage (I)	T_2_ (SD)	6.18	30.90	32.86	58.63	49.67	8.10	12.12	11.55
		T_3_ (MD)	6.33	32.69	33.1	58.23	49.38	7.80	10.44	9.82
		T_4_ (LD)	7.87	34.89	30.33	61.25	52.53	7.60	7.36	6.69
	Flowering to fruit set stage (II)	T_5_ (SD)	6.75	34.1	22.97	52.52	55.38	7.80	8.97	8.36
		T_6_ (MD)	6.02	33.93	28.4	56.58	73.04	7.90	7.62	6.93
		T_7_ (LD)	7.55	35.14	24.87	58.85	63.64	7.60	8.26	7.57
	Fruit growth stage (III)	T_8_ (SD)	6.52	31.47	27.26	52.36	67.96	8.80	11.28	10.62
		T_9_ (MD)	7.47	47.78	27.51	54.36	65.31	8.40	10.32	9.62
		T_10_ (LD)	7.45	42.08	24.69	55.21	66.89	8.20	13.22	12.48
	Fruit maturity stage (IV)	T_11_ (SD)	6.72	32.19	30.82	52.32	50.48	9.20	14.59	13.86
		T_12_ (MD)	6.78	40.15	31.08	55.63	58.17	9.50	14.28	13.54
		T_13_ (LD)	6.53	40.41	31.82	56.78	61.82	9.30	13.69	12.95
2007		T_1_ (CK)	4.53	28.26	70.68	32.7	54.9	6.24	9.19	8.45
	Bud burst to leafing stage (I)	T_2_ (SD)	4.32	27.86	73.25	40.65	70.08	7.52	10.61	10.04
		T_3_ (MD)	5.52	28.61	73.54	39.73	55.71	7.42	10.24	9.62
		T_4_ (LD)	6.37	27.14	76.25	45.84	45.62	6.80	8.23	7.56
	Flowering to fruit set stage (II)	T_5_ (SD)	5.5	29.29	73.99	40.04	58.36	7.65	7.6	6.99
		T_6_ (MD)	4.9	30.73	74.81	42.48	79.48	7.51	7.99	7.30
		T_7_ (LD)	5.35	25.43	77.42	42.48	65.64	7.23	8.94	8.24
	Fruit maturity stage (IV)	T_11_ (SD)	5.48	26.83	74.98	44.01	86.86	8.86	11.01	9.47
		T_12_ (MD)	5.32	28.52	76.12	45.65	77.95	8.45	10.58	9.12
		T_13_ (LD)	5.27	28.64	78.25	42.23	82.5	8.24	13.52	11.79

The greater the better index:

(2) $$r_{\mathrm{ij}}=\frac{\;x_{\mathrm{ij}}-x_{i}^{\min}}{x_{i}^{\max}-x_{i}^{\min}}a+b$$

The smaller the better index:

(3) $$r_{\mathrm{ij}}=\frac{\;x_{i}^{\max}-x_{\mathrm{ij}}}{\;x_{i}^{\max}-x_{i}^{\min}}a+b$$

where, *r*_*ij*_ is the relative membership degree with *j* as scheme and *i* as indicator relative to the excellent; $x_{i}^{\min}$ and $x_{i}^{\max}$ are the maximum and minimum values to the indicator among all the schemes respectively; *a* and *b* are coefficients to improve the formula and according to the actual situation and the model set, here given as *a* = 0.99, *b* = 0.01.

According to the membership degree function as described in Equation (1), the indicator value can be translated into the relative membership degree matrix:

(4) $$R=\left(r_{\mathrm{ij}}\right)$$

#### 2.3.3 Optimal fuzzy partition matrix

If the maximum value of each line in Equation (4) was extracted and named as the ideally excellent scheme:

(5) $$r_{p}=\overset{n}{\underset{j=1}{\vee }}r_{\mathrm{ij}}=\left(1,1,\ldots ,1\right)$$

And the minimum value of each line in equation (4) was also extracted and named as the ideally inferior scheme:

(6) $$r_{a}=\overset{n}{\underset{j=1}{\wedge }}r_{\mathrm{ij}}=\left(0,0,\ldots ,0\right)$$

According to the definition of the relative membership degree, the excellent and inferior schemes are the two poles of the reference system respectively. Then any one of the schemes belongs to the excellent or the inferior scheme with a certain membership degree of *u*_*pj*_ and *u*_*aj*_, that is, the excellent and inferior membership degrees, then the optimal fuzzy partition matrix is:

(7) $$U={\left[\begin{array}{cccc} U_{p1}, & U_{p2}, & \ldots & U_{\mathrm{pn}}\\ U_{a1}, & U_{a2} & \ldots & U_{\text{an}} \end{array}\right]}_{\left(2\times n\right)}$$

where, 0 ≤ *u*_*pj*_ ≤1, 0 ≤ *u*_*aj*_ ≤1, *u*_*pj*_ + *u*_*aj*_ = 1, *j* = 1, 2, …, *n*.

#### 2.3.4 Fuzzy evaluation model

Suppose that weighted‐vector of the evaluation system is given as

(8) $$\lambda =\left(\lambda_{1},\lambda_{2},\ldots ,\lambda_{m}\right)$$

where, $\sum \lambda_{i}=1$.

According to the least criterion of sum of squares with weight method for Euclid optimum and inferior distance, when *P* = 2, the objective function for the optimal value to relative optimal membership degree *u*_*kj*_ of j scheme is given by

(9) $$\min\left\{F=u_{\mathrm{pj}}^{2}\sum {\left[\lambda_{i}\left(r_{\mathrm{ij}}-r_{\mathrm{pj}}\right)\right]}^{2}+{\left(1-u_{\mathrm{pj}}\right)}^{2}\sum {\left[\lambda_{i}\left(r_{\mathrm{ij}}-r_{\mathrm{aj}}\right)\right]}^{2}\right\}$$

where Euclid optimum distance with weight method for *j* scheme is given as

(10) $$S_{\mathrm{pj}}=U_{\mathrm{pj}}\sqrt{\sum_{i=1}^{m}{\left[\lambda_{i}\left(r_{\mathrm{ij}}-r_{\mathrm{pj}}\right)\right]}^{2}}$$

And the Euclid inferior distance with weight method for *j* scheme is given by

(11) $$S_{\mathrm{aj}}=U_{\mathrm{aj}}\sqrt{\sum_{i=1}^{m}{\left[\lambda_{i}\left(r_{\mathrm{ij}}-r_{\mathrm{aj}}\right)\right]}^{2}}$$

To equation (9), derivation and let it equal to zero, then the RDI comprehensive benefits fuzzy evaluation model is:

(12) $$U_{\mathrm{kj}}=\sum_{i=1}^{m}\left[\lambda_{i}{\left(r_{\mathrm{ij}}-r_{\mathrm{aj}}\right)}^{2}\right]/\left\{\sum_{i=1}^{m}\left[\lambda_{i}{\left(r_{\mathrm{ij}}-r_{\mathrm{pj}}\right)}^{2}\right]+\sum_{i=1}^{m}{\left[\lambda_{i}\left(r_{\mathrm{ij}}-r_{\mathrm{aj}}\right)\right]}^{2}\right\}={\left\{1+\sum_{i=1}^{m}\left[\lambda_{i}{\left(1-r_{\mathrm{ij}}\right)}^{2}\right]/\sum_{i=1}^{m}\left[{\left(\lambda_{i}r_{\mathrm{ij}}\right)}^{2}\right]\right\}}^{-1}$$

where *U*_*kj*_ is an optimal membership degree for decision‐making; $\lambda_{i}$ is the weight of parameter *i* of RDI scheme *j*, $\lambda_{i}$ depends on the entropy weight and experts evaluation grade. Then according to the experimental data, the relative optimal membership degree *U*_*kj*_ can be calculated as Equation (12) and sorted the order, the greater *U*_*kj*_, the RDI scheme was more close to the best scheme relatively, namely, the RDI scheme with the greatest *U*_*kj*_ is the ideally best scheme.

### 2.4 Determination of the weight for information entropy of parameters

#### 2.4.1 Entropy‐weight method

In evaluation problem, the entropy to the *i*th index is defined as (Qiu, 2002):

(13) $$H_{i}=-k\sum_{j=1}^{n}f_{\mathrm{ij}}\ln f_{\mathrm{ij}}\;i=1,2,\ldots ,m$$

where, *f*_*ij*_ = $r_{\mathrm{ij}}/\sum_{j=1}^{n}r_{\mathrm{ij}}$, *k* = 1/ln *n*, *m* is the number of parameters, *n* is the number of the assessment objects.

In Equation (13), when *f*_*ij*_ = 0, then $f_{\mathrm{ij}}\ln f_{\mathrm{ij}}$ = 0, then

(14) $$\omega_{\mathrm{bi}}=\frac{1-H_{i}}{m-\sum_{i=1}^{m}H_{i}}$$

where 0 ≤ *ω*_*i*_ ≤ 1, $\sum_{i=1}^{m}\omega_{i}=1$, *ω*_*bi*_ is the weight of information entropy for the *i*th assessment index.

#### 2.4.2 Determination of the comprehensive weight for evaluation parameters

Comprehensive weight for evaluation index is the combination of the information entropy weight (objective weight) and the expert evaluation weight (subjective weight). The objective weight indicates the contribution degree of every specific data to the scheme set, however, after normalization, the subjective weight reflects the influence of experiences, which were gained by 10 experts who point out their authority weights on every evaluation index.

The comprehensive weight of the *i*th index is given by the following:

(15) $$\omega_{i}=\frac{\omega_{\mathrm{ai}}\cdot \omega_{\mathrm{bi}}}{\sum_{k=1}^{m}\omega_{\mathrm{ak}}\cdot \omega_{\mathrm{bk}}}$$

where *ω*_*ai*_ is the subjective weight of the *i*th assessment index, *ω*_*bi*_ is the information entropy weight of the *i*th assessment index, and 0 ≤ *ω*_*ai*_ ≤1, 0 ≤ *ω*_*bi*_ ≤1, i = 1,2, …, *m*.

### 2.5 Optimization of the analytic hierarchy model for multipurpose level

Suppose that the frame of the integration benefit evaluation hierarchy model can be divided into *M* levels (in this paper *M* = 3), and the highest level is *M*; If the number of the total evaluation parameters is *m* (in this paper *m* = 15), the specific optimization method can be expressed as follows (Ebel et al., 2001): Firstly, the basic matrix should be normalized using Equations (2) or (3) to establish the membership degree matrix in which each index is relatively optimal in the scheme; secondly, the information entropy weight and the expert evaluation weight should be calculated by Equation (14), and the comprehensive weight of *i*th index should also be calculated by Equation (13); thirdly, the comprehensive weight and the element of the degree matrix should be put into Equation (12) to get the level's relative optimal membership degree matrix, that is, the basic indicators of fuzzy matrix of the layer 2:

(16) U=Up11,Up12,…,Upn1⋮⋮⋮⋮Uam1,Uam2,…,Uanm

Fourthly, repeat the first to third step, and get the optimal membership degree matrix of the higher and the highest level until the end of the *M*, and then get the output of the highest level system, which is the decision or optimal membership degree vector *U*_*k*_ of the scheme *j*:

(17) $$U_{k}=\left(U_{1},\;U_{2},\;\ldots ,\;U_{n}\right)$$

From the value of the optimal membership degree vector in the formula (17), the best RDI scheme will be optimized.

## 3 RESULTS

According to the field experiment data during 2005–2007 on pear‐jujube tree at different growth stages, the analytic hierarchy model for multi‐purpose level based on information entropy was used to analyze and evaluate the comprehensive benefits. The 7 parameters on water use and yield and 8 parameters on fruit quality and economic benefit were used in the model and are listed in Table 3.

**TABLE 3:** Relative membership degree to the indices of regulated deficit irrigation in different growth stages of pear‐jujube tree.

Year	Growth stages	Treatment	*x*_1_	*x*_2_	*x*_3_	*x*_4_	*x*_5_	*x*_6_	*x*_7_
2005		T_1_ (CK)	0.010	0.010	0.641	0.175	0.139	0.358	0.183
	Bud burst to leafing stage (I)	T_2_ (SD)	1.000	1.000	0.941	0.450	0.354	0.963	0.946
		T_3_ (MD)	0.513	0.505	0.931	0.340	0.268	0.770	0.622
		T_4_ (LD)	0.338	0.340	0.389	0.560	0.440	0.221	0.121
	Flowering to fruit set stage (II)	T_5_ (SD)	0.936	1.000	0.010	0.010	0.010	0.010	0.072
		T_6_ (MD)	0.499	0.505	0.463	0.285	0.225	0.330	0.246
		T_7_ (LD)	0.279	0.340	0.246	0.450	0.354	0.078	0.010
	Fruit growth stage (III)	T_8_ (SD)	0.960	1.000	0.222	0.615	0.483	0.227	0.270
		T_9_ (MD)	0.503	0.505	0.271	0.725	0.570	0.156	0.092
		T_10_ (LD)	0.280	0.340	0.759	0.835	0.656	0.538	0.404
	Fruit maturity stage (IV)	T_11_ (SD)	0.943	1.000	1.000	0.780	0.613	1.000	1.000
		T_12_ (MD)	0.489	0.505	0.900	1.000	0.785	0.733	0.597
		T_13_ (LD)	0.326	0.340	0.813	0.835	0.656	0.600	0.444
	Entropy assessment		0.499	0.501	0.330	0.335	0.335	0.511	0.490
	Recombination assessment		0.324	0.175	0.183	0.102	0.049	0.306	0.196
2006		T_1_ (CK)	0.010	0.010	0.396	0.089	0.089	0.183	0.119
	Bud burst to leafing stage (I)	T_2_ (SD)	0.737	0.406	1.000	0.010	0.010	1.000	1.000
		T_3_ (MD)	0.378	0.208	0.649	0.208	0.327	0.247	0.462
		T_4_ (LD)	0.252	0.142	0.072	0.327	0.723	0.024	0.010
	Flowering to fruit set stage (II)	T_5_ (SD)	0.704	0.406	0.325	0.129	0.129	0.398	0.448
		T_6_ (MD)	0.375	0.208	0.036	0.208	0.208	0.034	0.033
		T_7_ (LD)	0.258	0.142	0.158	0.248	0.248	0.090	0.065
	Fruit growth stage (III)	T_8_ (SD)	0.744	0.406	0.166	0.406	0.406	0.279	0.319
		T_9_ (MD)	0.364	0.208	0.010	0.485	0.485	0.010	0.015
		T_10_ (LD)	0.265	0.142	0.409	0.604	0.604	0.283	0.233
	Fruit maturity stage (IV)	T_11_ (SD)	0.679	0.406	0.597	0.644	0.644	0.620	0.671
		T_12_ (MD)	0.369	0.208	0.580	0.525	0.525	0.402	0.415
		T_13_ (LD)	0.255	0.142	0.434	0.802	0.802	0.299	0.250
	Entropy assessment		0.515	0.485	0.370	0.389	0.241	0.504	0.496
	Recombination assessment		0.335	0.170	0.175	0.100	0.052	0.302	0.198
2007		T_1_ (CK)	0.010	0.010	0.628	0.010	0.010	0.378	0.041
	Bud burst to leafing stage (I)	T_2_ (SD)	0.521	0.670	1.000	0.129	0.129	0.997	0.765
		T_3_ (MD)	0.114	0.340	0.865	0.208	0.208	0.643	0.353
		T_4_ (LD)	0.132	0.230	0.256	0.327	0.327	0.091	0.010
	Flowering to fruit set stage (II)	T_5_ (SD)	0.668	0.670	0.010	0.604	0.604	0.078	0.201
		T_6_ (MD)	0.201	0.340	0.136	0.525	0.525	0.010	0.022
		T_7_ (LD)	0.211	0.230	0.353	0.683	0.683	0.215	0.051
	Fruit maturity stage (IV)	T_11_ (SD)	0.411	0.670	0.686	0.762	0.762	0.626	0.586
		T_12_ (MD)	0.268	0.340	0.775	0.921	0.921	0.637	0.312
		T_13_ (LD)	0.439	0.230	0.486	1.000	1.000	0.446	0.108
	Entropy assessment		0.488	0.512	0.271	0.339	0.390	0.514	0.486
	Recombination assessment		0.311	0.183	0.182	0.104	0.049	0.298	0.201
2005–2007	Expert assessment		0.650	0.350	0.550	0.300	0.150	0.600	0.400

Year	Growth stages	Treatment	*x*_8_	*x*_9_	*x*_10_	*x*_11_	*x*_12_	*x*_13_	*x*_14_	*x*_15_
2005		T_1_ (CK)	0.010	0.121	0.520	3.537	0.010	0.010	0.336	0.325

Bud burst to leafing stage (I)	T_2_ (SD)	0.337	0.247	0.010	3.410	0.014	0.293	0.513	0.525

	T_3_ (MD)	0.871	0.257	0.280	3.612	0.385	0.199	0.497	0.503

T_4_ (LD)	1.011	0.099	0.549	3.291	0.734	0.057	0.239	0.236

Flowering to fruit set stage (II	T_5_ (SD)	0.611	0.240	0.694	2.967	1.000	0.529	0.010	0.010

	T_6_ (MD)	0.664	0.076	1.000	3.218	0.579	0.387	0.256	0.250

T_7_ (LD)	0.640	0.115	0.593	3.703	0.385	0.151	0.158	0.150

Fruit growth stage (III)	T_8_ (SD)	0.765	0.010	0.702	2.856	0.989	0.811	0.439	0.439

	T_9_ (MD)	0.732	0.517	0.544	2.872	0.081	0.670	0.483	0.480

T_10_ (LD)	0.717	1.000	0.640	3.057	0.816	0.764	0.837	0.834

Fruit maturity stage (IV)	T_11_ (SD)	0.683	0.535	0.424	2.713	0.490	0.859	1.000	1.000

	T_12_ (MD)	0.876	0.481	0.334	2.928	0.636	0.953	0.958	0.957

T_13_ (LD)	0.919	0.516	0.334	3.169	0.339	1.000	0.876	0.872

Entropy assessment		0.191	0.128	0.183	0.219	0.142	0.138	0.509	0.475

Recombination assessment		0.025	0.015	0.023	0.028	0.041	0.036	0.200	0.300
2006		T_1_ (CK)	0.010	0.066	0.620	0.191	0.022	0.010	0.272	0.264
	Bud burst to leafing stage (I)	T_2_ (SD)	0.294	0.010	0.977	0.300	0.021	0.397	0.662	0.681
		T_3_ (MD)	0.357	0.115	1.000	0.345	0.010	0.268	0.432	0.442
		T_4_ (LD)	1.000	0.244	0.729	0.010	0.135	0.182	0.010	0.010
	Flowering to fruit set stage (II)	T_5_ (SD)	0.532	0.198	0.010	0.978	0.248	0.268	0.231	0.240
		T_6_ (MD)	0.227	0.188	0.541	0.528	0.948	0.311	0.045	0.043
		T_7_ (LD)	0.866	0.259	0.196	0.276	0.575	0.182	0.134	0.131
	Fruit growth stage (III)	T_8_ (SD)	0.436	0.043	0.429	0.996	0.746	0.699	0.547	0.553
		T_9_ (MD)	0.833	1.000	0.454	0.774	0.641	0.527	0.414	0.415
		T_10_ (LD)	0.825	0.666	0.178	0.680	0.704	0.440	0.812	0.809
	Fruit maturity stage (IV)	T_11_ (SD)	0.520	0.086	0.777	1.000	0.054	0.871	1.000	1.000
		T_12_ (MD)	0.545	0.553	0.803	0.633	0.358	1.000	0.957	0.956
		T_13_ (LD)	0.440	0.568	0.875	0.506	0.503	0.914	0.877	0.875
	Entropy assessment		0.161	0.260	0.157	0.156	0.118	0.147	0.500	0.500
	Recombination assessment		0.028	0.013	0.024	0.028	0.035	0.038	0.200	0.300
2007		T_1_ (CK)	0.113	0.635	0.791	0.010	0.219	0.010	0.228	0.213
	Bud burst to leafing stage (I)	T_2_ (SD)	0.010	0.560	0.526	0.558	0.559	0.494	0.423	0.436
		T_3_ (MD)	0.588	0.700	0.495	0.495	0.237	0.456	0.372	0.377
		T_4_ (LD)	0.998	0.427	0.216	0.916	0.010	0.222	0.096	0.090
	Flowering to fruit set stage (II)	T_5_ (SD)	0.580	0.829	0.449	0.516	0.296	0.543	0.010	0.010
		T_6_ (MD)	0.290	1.096	0.365	0.684	0.770	0.490	0.063	0.054
		T_7_ (LD)	0.507	0.106	0.096	0.684	0.460	0.384	0.193	0.185
	Fruit maturity stage (IV)	T_11_ (SD)	0.572	0.368	0.348	0.790	0.936	1.000	1.000	1.000
		T_12_ (MD)	0.493	0.684	0.230	0.903	0.736	0.845	0.949	0.947
		T_13_ (LD)	0.469	0.707	0.010	0.667	0.838	0.766	0.893	0.890
	Entropy assessment		0.169	0.155	0.161	0.181	0.160	0.173	0.500	0.500
	Recombination assessment		0.029	0.008	0.019	0.027	0.048	0.045	0.201	0.298
2005–2007	Expert assessment		0.150	0.100	0.130	0.150	0.250	0.220	0.400	0.600

### 3.1 Entropy weight of parameters and relatively optimal membership degree of second level

According to the optimization method described in Section 2.5, the entropy weight of 7 parameters on water use and yield and 8 parameters on fruit quality and economic benefit were calculated and listed in Table 4. The optimal membership degree for decision‐making were calculated as described in Section 2.3.4. and the comprehensive weight for each evaluation parameters were calculated following the description in Section 2.4.2, then the optimal membership degree of the parameters in the second level were obtained (Table 5).

**TABLE 4:** Optimal membership degree for the indices in the second layer.

Year	Growth stages	Treatment	Water‐saving indexes	Yield indexes	Water use efficiency (WUE)	Quality indexes	Economy benefit (EB)
2005		T_1_ (CK)	0.010	0.584	0.179	0.162	0.201
	Bud burst to leafing stage (I)	T_2_ (SD)	1.000	0.900	0.998	0.268	0.548
		T_3_ (MD)	0.527	0.867	0.872	0.171	0.507
		T_4_ (LD)	0.215	0.371	0.066	0.274	0.097
	Flowering to fruit set stage (II)	T_5_ (SD)	0.997	0.063	0.012	0.701	0.010
		T_6_ (MD)	0.505	0.391	0.172	0.371	0.111
		T_7_ (LD)	0.156	0.184	0.015	0.121	0.041
	Fruit growth stage (III)	T_8_ (SD)	0.999	0.213	0.100	0.799	0.386
		T_9_ (MD)	0.512	0.294	0.035	0.395	0.468
		T_10_ (LD)	0.157	0.875	0.503	0.787	0.963
	Fruit maturity stage (IV)	T_11_ (SD)	0.997	0.954	1.000	0.757	1.000
		T_12_ (MD)	0.490	0.934	0.834	0.768	0.998
		T_13_ (LD)	0.202	0.906	0.610	0.590	0.980
	Entropy assessment		0.203	0.212	0.177	0.215	0.193
	Recombination assessment		0.030	0.047	0.041	0.054	0.029
2006		T_1_ (CK)	0.010	0.280	0.048	0.215	0.126
	Bud burst to leafing stage (I)	T_2_ (SD)	0.788	0.774	1.000	0.263	0.814
		T_3_ (MD)	0.228	0.588	0.189	0.192	0.385
		T_4_ (LD)	0.093	0.053	0.010	0.034	0.010
	Flowering to fruit set stage (II)	T_5_ (SD)	0.754	0.213	0.339	0.324	0.097
		T_6_ (MD)	0.224	0.070	0.011	0.597	0.012
		T_7_ (LD)	0.098	0.107	0.018	0.198	0.032
	Fruit growth stage (III)	T_8_ (SD)	0.795	0.133	0.153	0.768	0.605
		T_9_ (MD)	0.211	0.095	0.010	0.578	0.341
		T_10_ (LD)	0.103	0.417	0.127	0.537	0.948
	Fruit maturity stage (IV)	T_11_ (SD)	0.725	0.679	0.754	0.611	1.000
		T_12_ (MD)	0.216	0.624	0.326	0.762	0.998
		T_13_ (LD)	0.095	0.515	0.145	0.797	0.980
	Entropy assessment		0.199	0.211	0.170	0.220	0.200
	Recombination assessment		0.030	0.047	0.039	0.055	0.030
2007		T_1_ (CK)	0.039	0.509	0.225	0.303	0.082
	Bud burst to leafing stage (I)	T_2_ (SD)	0.408	0.812	0.989	0.627	0.372
		T_3_ (MD)	0.113	0.786	0.694	0.382	0.273
		T_4_ (LD)	0.090	0.168	0.041	0.165	0.020
	Flowering to fruit set stage (II)	T_5_ (SD)	0.525	0.119	0.079	0.445	0.010
		T_6_ (MD)	0.133	0.141	0.033	0.701	0.014
		T_7_ (LD)	0.109	0.376	0.095	0.442	0.060
	Fruit maturity stage (IV)	T_11_ (SD)	0.866	0.233	0.722	0.729	0.307
		T_12_ (MD)	0.596	0.128	0.138	0.305	0.224
		T_13_ (LD)	0.487	0.719	0.705	0.605	0.869
	Entropy assessment		0.198	0.209	0.192	0.219	0.181
	Recombination assessment		0.030	0.046	0.044	0.055	0.027
2005–2007	Expert assessment		0.150	0.220	0.230	0.250	0.150

**TABLE 5:** Optimal Euclidean distance and integrated compositor of regulated deficit irrigation in different growth stages of pear‐jujube tree.

Growth stages	Year	Treatment	*U*_*kj*_	Compositor	Year	Treatment	*U*_*kj*_	Compositor	Year	Treatment	*U*_*kj*_	Compositor
	2005	T_1_ (CK)	0.154	11	2006	T_1_ (CK)	0.052	11	2007	T_1_ (CK)	0.169	9
Bud burst to leafing stage (I)		T_2_ (SD)	0.733	5		T_2_ (SD)	0.696	2		T_2_ (SD)	0.812	3
	
T_3_ (MD)	0.584	6		T_3_ (MD)	0.206	8		T_3_ (MD)	0.523	7
	
T_4_ (LD)	0.102	12		T_4_ (LD)	0.002	13		T_4_ (LD)	0.022	13
Flowering to fruit set stage (II)		T_5_ (SD)	0.338	8		T_5_ (SD)	0.209	7		T_5_ (SD)	0.146	11
	
T_6_ (MD)	0.205	9		T_6_ (MD)	0.177	10		T_6_ (MD)	0.233	8
	
T_7_ (LD)	0.020	13		T_7_ (LD)	0.022	12		T_7_ (LD)	0.163	10
Fruit growth stage (III)		T_8_ (SD)	0.491	7		T_8_ (SD)	0.472	5		T_8_ (SD)	0.635	6
	
T_9_ (MD)	0.205	10		T_9_ (MD)	0.198	9		T_9_ (MD)	0.121	12
	
T_10_ (LD)	0.792	3		T_10_ (LD)	0.390	6		T_10_ (LD)	0.789	4
Fruit maturity stage (IV)		T_11_ (SD)	0.976	1		T_11_ (SD)	0.838	1		T_11_ (SD)	0.907	1
	
T_12_ (MD)	0.928	2		T_12_ (MD)	0.683	3		T_12_ (MD)	0.849	2
	
T_13_ (LD)	0.760	4		T_13_ (LD)	0.578	4		T_13_ (LD)	0.679	5

### 3.2 Entropy weight of parameters and relatively optimal membership degree of first level

According to the optimization method described in Section 3.1, the entropy weight of 5 parameters of second level (Table 5) were calculated. It showed that the comprehensive benefits under different water deficit degree at different growth stages were very notable. As for the RDI scheme with higher integrative benefits, i.e., the optimal membership degree above 0.80, in 2005, the order for different RDI treatments was: IV_SD_ > IV_MD_ > III_LD_ > IV_LD_ > I_MD_; and in 2006 the order was: IV_SD_ > IV_MD_ > IV_LD_ > I_MD_ + IV_SD_; However, the order in 2007 was: I_SD_ + IV_MD_ > IV_SD_ > I_MD_ + IV_LD_ > IV_MD_ > IV_LD_, which indicated that double‐stage water deficit scheme gained better comprehensive benefits under conditions of more rainwater in fruit growth stage. However, as for the RDI scheme with higher integrative benefits, i.e., the optimal membership degree between 0.50 and 0.80, in 2005 and 2006 the order were III_SD_ > I_SD_ > III_MD_ andI_LD_ + IV_MD_ > I_SD_ > III_LD_ > III_SD_ respectively, in 2007 it was I_MD_ > II_SD_ > II_LD_. As for the low integrative benefits scheme with the optimal membership degree less than 0.50, the order in 2005 to 2007 in turn to: II_MD_ > I_LD_ > I_SD_ > CK > II_LD_, II_SD_ > I_MD_ > II_LD_ > III_MD_ > I_LD_ > II_MD_ > CK and III_MD_ > I_LD_ > II_MD_ > CK respectively.

The results in 2005–2006 showed that the RDIIB under single‐stage water deficit at fruit maturity stage were better than the other treatments, and the best RDIIB was gained under moderate (IV_SD_) or severe deficit (IV_MD_) at fruit maturity stage. The results in 2006–2007 also indicated that the four double‐stage water deficit scheme have the better RDIIB, and the best scheme was severe water deficit at bud burst to leafing stage plus moderate water deficit at fruit maturity stage.

## 4 DISCUSSION

It is the main task to establish the model of water‐yield quality and optimize the water‐saving and efficient irrigation pattern in arid areas (Kang et al., 2007), the traditional water stress is a passive adaptation of plants under water scarcity, but the efficient water‐saving irrigation for improving fruit quality is an active irrigation controlling for better quality and higher benefit. The benefits of water stress in given stage (e.g., Stage I, shoot growth reduced with less influence to fruit growth) was tested in many experiments such as pear and peach (Paul & Goodwin, 2003). In most of the references, the improvement of fruit yield is inconsistent with the fruit quality, can we gain higher fruit quality under acceptable higher yield? Which quality index is more important for higher economic benefit and can we find a scientific integrative quality parameter? Fuzzy evaluation about integrated benefit of RDI may be a possible key to analyze the large number of data on water use, fruit yield, fruit quality, economic benefits, and so on. The evaluation result may be more scientific and comprehensive than other evaluation method with only some single parameters.

Theory of information entropy has been applied in many subjects such as physics, chemistry, biology, and economic management since its naissance (Fang et al., 2001). In this theory, if the information entropy of a certain parameter was more smaller, it indicate has a greater degree of variation, so does the bigger information was been provided, the role it has made in the comprehensive evaluation and its weight was also more greater. Vice versa, the bigger information entropy means less information and smaller weight in the evaluation system. Therefore, according to the degree of variation of each parameter, the information entropy can be used to calculate the weight of every factor, which provide objective basis for multi‐criteria comprehensive evaluation (Tang et al., 2000). In this paper, Comprehensive weight index of the RDIIB evaluation includes subjective and objective evaluation method; however, both of them have their advantages and disadvantages. RDIIB include various information about water‐saving, yield, WUE, EB and so on, the variety of the benefit's factors bring many difficulties to the comprehensive evaluation, thus in the comprehensive evaluation, both the opinion of the experts and the inherent character of the index should be fully considered, the entropy theory method is a better combination of the subjective and objective approach to comprehensively evaluate the benefits of the program, which avoid the defects of only application of subject or object method.

RDI research on nut crops such as almond showed that it decreased kern yields by about 10%–20% but improved WUE and water savings up to 50% as compared to full irrigation (Romero et al., 2004); However, experiment on apple indicated that RDI decrease yield and fruit size but improve WUE and fruit quality irrespective of the timing of application (Ebel et al., 2001; Mpelasoka et al., 2001). Similarly, the study in this paper shows that different RDI schemes have an obliviously different effect on water consumption, yield, WUE, EB and so on. Therefore, comprehensive evaluation on different RDI scheme is very important for the optimization of the best RDI pattern to improve the total economic benefit of irrigation control. Furthermore, Zhang and Huang (2008) investigated the optimized irrigation regime of RDI on spring wheat in an arid environment of Northwest China, in which the average grain yield, water use efficiency (WUE), water supply use efficiency (WUEs), and the integrated nutrient index (INI) for two years data were used as factors to establish the single‐factor evaluation matrix for integrative evaluation of RDI regimes. The results showed that heavy water stress at elongation stage and full irrigation at booting and heading stage is the better RDI pattern which has the highest comprehensive evaluation index, and it should be recommended to be the most optimal RDI scheme for the spring wheat in the experiment area. Furthermore, in this paper, it was also found that the RDI scheme which has a severe or moderate single‐stage water deficit at fruit mature stage, or severe water stress during bud burst to leafing stage combining moderate stress at fruit mature stage has the best RDIIB. Certainly, the multiphase of RDI data is not systemic during different years in our field experiments, so the result has some certain limitations. Thus, in the future, it is essential to establish more comprehensive multiphase RDI schemes and compare the result of the different years, and then the results will be more comprehensive and accurate for comprehensive benefit evaluation.

## 5 CONCLUSIONS

In summary, the analytic hierarchy model for multi‐purpose level evaluated that the single‐stage water deficit treatments with moderate or severe in fruit maturity have the higher RDIIB, and the double‐stage water deficit treatment with severe stress at bud burst to leafing stage combining moderate water deficit at fruit mature stage was also an acceptable RDI scheme in case of more water at fruit growth stage. In the fuzzy comprehensive evaluation, the determination of the weight has a significant influence to the results of the evaluation. We get the comprehensive weight on the base of combination between the information entropy and the expert method, which can enhance the reliability of the weight and increase the accuracy of the evaluation results.

## CONFLICT OF INTEREST

We declare that we do not have any commercial or associative interest that represents a conflict of interest in connection with the work submitted.

## Data Availability

Data sharing is not applicable to this article as no new data were created or analyzed in this study.
